# KI in der Rehabilitation – Anwendung künstlicher mentaler Modelle für eine personalisierte Medizin

**DOI:** 10.1007/s00103-025-04090-w

**Published:** 2025-07-04

**Authors:** Sabine Janzen, Prajvi Saxena, Cicy Agnes, Wolfgang Maaß

**Affiliations:** Smart Service Engineering, Deutsches Forschungszentrum für Künstliche Intelligenz (DFKI), Stuhlsatzenhausweg 3, 66123 Saarbrücken, Deutschland

**Keywords:** Knierehabilitation, Psychologische Faktoren, Personalisierte Therapieplanung, Sprachmodelle (LLMs), Datengestützte Entscheidungsunterstützung, Knee rehabilitation, Psychological factors, Personalized therapy planning, Large Language Models (LLMs), Data-driven decision support

## Abstract

Künstliche Intelligenz (KI) kann in der Prävention und Rehabilitation eine patient*innenzentrierte Versorgung unterstützen. In Deutschland wurden 2023 fast 1,9 Mio. Patient*innen in Rehaeinrichtungen behandelt, viele aufgrund von Erkrankungen des Muskel-Skelett-Systems. Der Erfolg einer Reha hängt von der Zusammenarbeit zwischen Patient*in, Arzt oder Ärztin und Therapeut*in sowie der aktiven Mitarbeit ab. Kognitive Einschränkungen, Sprachbarrieren und psychische Faktoren erschweren jedoch die Entscheidungsfindung und Kommunikation vieler Patient*innen. Dies führt zu unvollständigen oder verzerrten Daten und beeinträchtigt eine individualisierte Therapie. Ein Lösungsansatz besteht im Einsatz künstlicher mentaler Modelle (KMM), die unbekannte mentale Modelle von Patient*innen antizipieren. Diese Konzepte basieren auf kognitionswissenschaftlichen Theorien und World Models aus der KI. KMM können Therapieentscheidungen optimieren, Fehleinschätzungen korrigieren und so den Rehaerfolg steigern. Beispielsweise in der Knierehabilitation kann ein KI-Agent ermitteln, wie Patient*innen ihre Genesung wahrnehmen, und individuelle Anpassungen ermöglichen. Das BMFTR-Projekt „FedWell“ erforscht den Einsatz von KMM in der Rehabilitation. Ein diskriminierungsfreies Basismodell wurde mithilfe von Daten aus Online-Foren, Nutzer*innenstudien und maschinellen Lernmodellen entwickelt. Erste Ergebnisse zeigen, dass KI-gestützte Modelle individuelle Annahmen und Erwartungen von Patient*innen im Rehaprozess vorhersagen und personalisierte Therapien ermöglichen können. Dieser Beitrag stellt das Forschungsdesign des Projekts vor und berichtet erste Ergebnisse der initialen Erhebungsphase.

## Abstract (en)

Artificial intelligence (AI) can support patient-centered care in prevention and rehabilitation. In Germany, almost 1.9 million patients were treated in rehabilitation hospitals in 2023, mostly due to musculoskeletal disorders. The success of rehabilitation depends on cooperation between patient, doctor, and therapist as well as active participation. However, cognitive limitations, language barriers, and psychological factors tackle decision-making and communication abilities of patients. This leads to incomplete or distorted data and impairs individualized therapy. A potential solution approach is to apply artificial mental models (AMMs) that anticipate patients’ unknown mental models. These concepts are based on cognitive science theories and world models from AI. AMMs can optimize treatment decisions, correct misjudgments, and thus increase the success of rehabilitation. Particularly in knee rehabilitation, an AI agent can determine how patients perceive their recovery and enable individual adjustments. The BMFTR project FedWELL investigates the use of AMM in rehabilitation. A non-discriminatory base model was developed using data from online forums, user studies, and machine learning models. Initial results show that AI-supported models can predict individual assumptions and expectations of patients within the rehabilitation process and enable personalized therapies. This article presents the research design of the project and reports the first results of the initial survey phase.

## Hintergrund

In Bereich der Prävention und Rehabilitation (Reha) können adaptive und personalisierte Systeme auf Basis von Methoden der künstlichen Intelligenz (KI) eine entscheidende Rolle als Unterstützer einer patient*innenzentrierten Versorgung spielen [1]. Im Jahr 2023 wurden 1.886.876 Patient*innen mit einer durchschnittlichen Verweildauer von 25,5 Tagen in einer Vorsorge- oder Rehabilitationseinrichtung in Deutschland behandelt.^1^ Der Großteil der vollstationären Patient*innen (ca. 550.000) wurde aufgrund von Krankheiten des Muskel-Skelett-Systems (z. B. Arthrose) sowie aufgrund von Verletzungen behandelt. Der Erfolg einer Rehabilitationsmaßnahme (Reha) hängt entscheidend von der guten Zusammenarbeit zwischen Patient*in, Arzt oder Ärztin und Physiotherapeut*in sowie der Bereitschaft der Patient*innen zur aktiven Mitarbeit in einem strukturierten, oft auch schmerzhaften Programm ab [2].

Doch Patient*innen weisen in Situationen, die von Krankheit, Schmerzen und medizinischen Entscheidungen geprägt sind, kognitive Einschränkungen auf. Dadurch sind eine informierte Entscheidungsfindung, das Verständnis komplexer medizinischer Sachverhalte und die wirksame Artikulation von Symptomen und individuellen Anliegen nicht immer möglich [3–6]. Bis zu 30 % der Patient*innen in Rehaeinrichtungen können als sprachlich eingeschränkt charakterisiert werden; d. h., sie sind wegen der genannten kognitiven Einschränkungen, Migrationshintergründen oder auch psychischer Probleme nicht in der Lage, sich entsprechend auszudrücken [7–10]. Zudem erleben Patient*innen in der Reha oft Zielkonflikte, die für Ärzt*innen, Physiotherapeut*innen und Psycholog*innen nicht sofort ersichtlich sind. So steht beispielsweise dem Wunsch, die Reha rasch und erfolgreich abzuschließen, mitunter das Bedürfnis nach Entlastung gegenüber, etwa bei Überforderung im häuslichen Umfeld [11, 12].

In Gänze führt dies zu einer Generierung unvollständiger, ungenauer oder verzerrter Daten, welche die Bedürfnisse und Umstände der Patient*innen nicht erfassen können [3–6]. Es fehlt an strukturierten und umfassenden Ansätzen zur Erhebung psychologischer Parameter in der Reha, die essenziell für eine ganzheitliche und patient*innenzentrierte Therapie sind. Ein potenzieller Ansatz besteht in der Anwendung von künstlichen mentalen Modellen in therapeutischen Kontexten und damit verbundener KI-Systeme zur Verbesserung der personalisierten Patient*innenversorgung und der Therapieergebnisse [13–15].

**Abb. 1:**
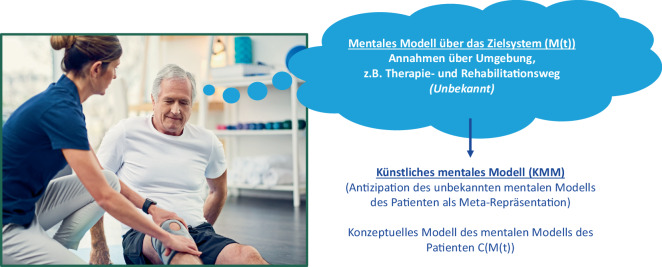
Zusammenhang zwischen dem mentalen Modell eines Patienten und einem künstlichen mentalen Modell im Kontext einer Rehabilitationssituation nach einer Knie-OP (Foto: iStock.com/Charday Penn)

Der Begriff des mentalen Modells entstammt der Kognitionswissenschaft. Mentale Modelle sind die kognitiven Rahmen, die Menschen verwenden, um ihre Umgebung, d. h. die Welt, die sie umgibt, zu verstehen und sich in ihr zurechtzufinden [16–19]. Das mentale Modell der zu therapierenden Person (Abb. 1) reflektiert deren Annahmen über das Zielsystem, d. h. die Umgebung, mit der sie interagiert, z. B. Annahmen über ihren Therapie- und Rehabilitationsweg [17, 20, 21]. Da das mentale Modell der Person implizit und darum unbekannt ist, besteht der Ansatz künstlicher mentaler Modelle (KMM) darin, ein konzeptuelles Modell ihres mentalen Modells zu kreieren, welches dieses in Form einer Metarepräsentation antizipiert.

Kognitionswissenschaftliche mentale Modelle weisen eine konzeptionelle Ähnlichkeit zu World Models in der KI auf [22]. Diese beziehen sich auf die interne Darstellung der Umgebung eines Systems, die es verwendet, um die Welt um es herum zu verstehen, vorherzusagen und mit ihr zu interagieren. Im Wesentlichen ermöglicht das World Model einer KI, mögliche Ergebnisse ihrer Handlungen zu simulieren, Veränderungen vorauszusehen und ihr Verhalten entsprechend anzupassen. Sowohl mentale Modelle als auch World Models dienen dem Zweck, die Außenwelt intern zu simulieren, um adaptives und zielorientiertes Verhalten zu ermöglichen. Mentale Modelle werden jedoch durch eine Kombination aus angeborenen Mechanismen, Lernen und sozialen Einflüssen geformt, während World Models explizit durch Daten und Algorithmen entworfen und trainiert werden. KMM lassen sich als World Models verstehen, die unbekannte, mentale Modelle von Patient*innen antizipieren. Eine Verwendung von KMM in der Reha kann die Wirksamkeit von Therapien fördern, Entscheidungsprozesse unterstützen und dazu beitragen, Wissenslücken und falsche Vorstellungen von Patient*innen zu erkennen und zu korrigieren [18].

Bestehende Forschungsarbeiten unterstreichen die Notwendigkeit, die mentalen Modelle der einzelnen Patient*innen genau zu erfassen und zu verstehen, insbesondere in Therapie- und Rehaszenarien [20, 23–25]. Beispielsweise nach Knieverletzungen oder -operationen ist dies essenziell, aber auch herausfordernd. Selbst kleine Einschränkungen beeinträchtigen die Mobilität erheblich. Nach Verletzungen wie Kreuzbandrissen oder Meniskusschäden müssen Patient*innen Kraft, Beweglichkeit und Stabilität schrittweise zurückgewinnen [26]. Ein langsamer Fortschritt ist dabei eine der größten Herausforderungen. Frustration kann entstehen, wenn Schmerzfreiheit oder volle Beweglichkeit nicht sofort erreicht werden. Entscheidend ist das richtige Maß: Überbelastung kann die Heilung stören, übermäßige Schonung führt zu Versteifung oder Muskelschwäche [27]. Zudem können Schmerzen die Motivation mindern, sodass Patient*innen wichtige Übungen meiden. Psychologische Faktoren, wie Angst vor einer erneuten Verletzung, spielen ebenfalls eine Rolle [28].

Das KMM der zu rehabilitierenden Person kann erfassen, wie diese ihre Verletzung, den Genesungsprozess und ihre Umgebung wahrnimmt.^2^ Das Modell verknüpft physische, psychologische und Umweltfaktoren. Physisch umfasst es den Zustand des Knies, das Schmerzlevel und die Bewegungseinschränkungen. Es erkennt Zusammenhänge zwischen Bewegungen und Schmerzen, z. B. dass Dehnen Steifheit verringert. Erwartungen hinsichtlich der Heilungsdauer werden ebenfalls berücksichtigt. Psychologisch integriert das KMM Überzeugungen zur Genesungsfähigkeit, die durch frühere Erfahrungen und das Vertrauen in den Rehaprozess beeinflusst werden. Optimistische Patient*innen sehen Fortschritt, während entmutigte Personen die Therapie als langwierig wahrnehmen. Umweltfaktoren wie familiäre Unterstützung oder berufliche Verpflichtungen fließen mit ein. Das KMM bildet Überzeugungen, Verhalten und Fortschritt des*der Patient*in ab. So kann es voraussagen, wie die Person auf Übungen reagieren wird und welche Ängste bestehen. Dies ermöglicht Therapeut*innen, personalisierte Strategien zu entwickeln, um die Motivation und den Rehaerfolg zu steigern.

Das vom Bundesministerium für Forschung, Technologie und Raumfahrt (BMFTR) geförderte Forschungsprojekt „FedWell“ (Life-Long Federated User and Mental Modeling for Health and Well-being) am Deutschen Forschungszentrum für Künstliche Intelligenz (DFKI) untersucht den Einsatz von KMM in KI-Systemen für das Gesundheitswesen. Es konzentriert sich auf 2 Anwendungsfälle: Reha nach Knieverletzungen und Therapieentscheidungen für Patient*innen mit eingeschränkter Entscheidungsfähigkeit (z. B. Demenz). FedWell entwickelt und evaluiert KMM-gestützte KI-Systeme für private und berufliche Anwendungen. Besonderer Wert wird auf Datenschutz gelegt: Die KMM laufen lokal bei Patient*innen oder Ärzt*innen, sodass keine sensiblen Daten zentral gespeichert werden. Dadurch bleibt die Privatsphäre gewahrt, während eine kontinuierliche Nutzung möglich ist. Um dies zu ermöglichen, werden Methoden zur Effizienzsteigerung wie Knowledge Destillation [29] genutzt, um KI-Modelle ressourcenschonend und leistungsfähig zu machen. Für das Basismodell eines KMM wurden Large Language Models (LLMs) eingesetzt. LLMs sind KI-Modelle, die mit großen Textdatenmengen trainiert wurden, um Sprache zu verstehen und zu generieren. Sie ermöglichen kontextbezogene Antworten, Textgenerierung und komplexe Analysen.

Dieser Beitrag erläutert das Forschungsdesign für die Untersuchung von KMM in KI-Systemen im Gesundheitswesen im Projekt FedWell und präsentiert erste Ergebnisse der initialen Erhebungsphase (Evaluierung des Basismodells).

## Forschungsdesign für die Untersuchung von künstlichen mentalen Modellen und Methoden in der Erhebungsphase

**Abb. 2:**
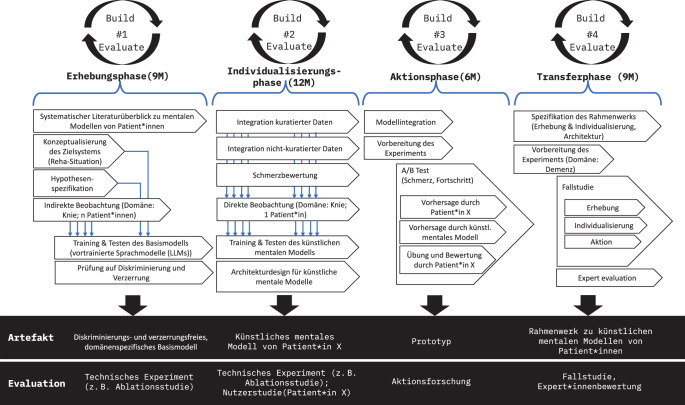
Forschungsdesign zur Untersuchung künstlicher mentaler Modelle (KMM) in KI-Systemen im Gesundheitswesen im Forschungsprojekt „FedWell“ am Deutschen Forschungszentrum für Künstliche Intelligenz (Abbildung aus [31], Rechte bleiben bei den Autor*innen)

Für die Untersuchung von KMM in KI-Systemen im Gesundheitswesen wurde im Projekt „FedWell“ ein Forschungsdesign in Orientierung an „Design Science“ spezifiziert [30–32]. Design Science ist ein forschungsorientierter Ansatz, der innovative Artefakte wie Modelle, Methoden oder Systeme entwickelt, um komplexe Probleme zu lösen. Das Forschungsdesign umfasst 4 iterativ angelegte Phasen: *Erhebung, Individualisierung, Aktion *und *Transfer *(Abb. 2). Das Vorgehen ist iterativ angelegt und umfasst Zyklen der Entwicklung, Evaluierung und Verfeinerung. Innerhalb jedes Zyklus wird ein Artefakt erstellt und evaluiert [33]. Dieses Artefakt kann ein Modell, eine Methode, ein Rahmenwerk oder ein technischer Prototyp sein. Für die Evaluation der Artefakte werden in den 4 Phasen verschiedene Methoden verwendet, z. B. technische Experimente, Nutzer*innenstudien, Prototyping, Action Research oder Fallstudien. Da der Schwerpunkt auf der Generierung neuen Wissens liegt, umfasst das Forschungsdesign nicht nur Erkenntnisse über das spezifische, problemlösende Artefakt, d. h. das KMM, das entwickelt wurde, sondern auch Beiträge zu gesetzten Theorien und Praktiken auf dem Gebiet in Form von Publikationen.

Im Folgenden liegt der Fokus auf dem Vorgehen in der Erhebungsphase, deren Ziel die Generierung eines diskriminierungs- und verzerrungsfreien, domänenspezifischen Basismodells eines KMM im Bereich der Kniereha ist (Abb. 2). Ein solches maschinelles Lernmodell benötigt große Mengen an Daten, um Muster, Zusammenhänge und Variationen in den Eingaben zu erkennen und eine Generalisierungsfähigkeit zu entwickeln. Aus diesem Grund umfasst die Erhebungsphase eine indirekte Beobachtung von Patient*innen zur Erstellung eines großen Datensatzes durch einen 2‑stufigen Ansatz bestehend aus systematischer Datenextrahierung (Data Scraping) und einer empirischen Nutzer*innenstudie (*n* = 116). Der resultierende Datensatz wird für das Training des KMM-Basismodells verwendet. Auftretende Halluzinationen^3^ und Verzerrungen im Basismodell werden behandelt. Das resultierende Artefakt – ein diskriminierungs- und verzerrungsfreies, domänenspezifisches KMM-Basismodell – wird in einem technischen Experiment evaluiert.

### Indirekte Beobachtung von Patient*innen

Für die indirekte Beobachtung wurde in FedWell in einem ersten Schritt eine Data-Scraping-Strategie aufgesetzt. Data Scraping bezeichnet den automatisierten Prozess des Extrahierens von Daten aus Webseiten oder anderen digitalen Quellen. Dabei wurden Programme wie „BeautifulSoup“ und „Scrapy“ genutzt, um strukturierte Informationen aus HTML-Seiten, wie z. B. „Physio.de“, zu extrahieren. Der Fokus lag hierbei auf Konversationen zwischen Patient*innen, Physiotherapeut*innen und Ärzt*innen und kuratierten Artikeln im Themenbereich Krankengymnastik, Therapiemethoden, Knieverletzungen, Rehabilitation, Knieoperation, Physiotherapie etc. Im Ergebnis wurden 7000 Nachrichtenartikel und 67.000 Konversationen extrahiert, die im Durchschnitt aus 8 „Turns“, d. h. einzelnen Äußerungen der Teilnehmer*innen des Gesprächs, bestanden. Der Großteil der Konversationen behandelte Themen rund um die Physiotherapie von Knie und Beinen (65 %). Darüber hinaus wurden spezifische Behandlungsmethoden (15 %) sowie Themen der Nachrichtenartikel diskutiert (21 %). Anschließend wurden die Daten vorverarbeitet, d. h., Sonderzeichen, Duplikate und leere Zeichenfolgen wurden entfernt sowie alle Sätze in Kleinbuchstaben dargestellt. Eine Filterung der Daten hinsichtlich ihrer Relevanz für die Domäne Kniereha fand in einem zweiten Schritt statt, in dem mit „Ereignisextraktion“ (Named Entity Recognition; [34]) sowie „Clustering“ Methoden der natürlichen Sprachverarbeitung eingesetzt wurden. Im Ergebnis wurde ein Datensatz (*n* = 4364) mit 2321 relevanten Konversationen und 2043 Artikeln spezifiziert, die Ereignisse oder Entitäten im Zusammenhang mit Knierehabilitation thematisierten und 4 Clustern zuzuordnen waren: Therapien, Krankheitsbild, Verlauf, Diagnoseverfahren.

Der zweite Teil der indirekten Beobachtung bestand in der Durchführung einer empirischen Studie mit 116 Teilnehmer*innen. Die Rekrutierung der Studienteilnehmer*innen erfolgte im Rahmen eines öffentlichen „Tages der offenen Tür“ an der Universität des Saarlandes in Saarbrücken im Mai 2024. Teilnehmende waren reguläre Besucher, überwiegend Eltern, deren Kinder parallel an einem betreuten Bastelangebot teilnahmen. Es handelte sich nicht um Patient*innen, sondern um freiwillige, nicht vorab ausgewählte Erwachsene, die ohne gezielte Ansprache an der Studie ohne besonderen Eingriffs- oder Belastungsgrad teilnahmen. Da die Studie keinen Eingriff in die körperliche oder psychische Integrität der Teilnehmenden beinhaltete und keinerlei besondere Belastung verursachte, war ein Ethikvotum gemäß den geltenden Richtlinien nicht erforderlich.

Ziel der Studie war es, die von den Teilnehmer*innen erwartete und tatsächlich wahrgenommene Anstrengung bei Sportübungen ihren Persönlichkeitsmerkmalen, ihrer medizinischen Vorgeschichte und den psychosozialen Faktoren gegenüberzustellen. Alle Teilnehmer*innen wurden während der Studie über die beabsichtigte Datennutzung für das Training eines KI-Modells am DFKI informiert. Die Teilnehmer*innen wurden gebeten, einen digitalen Fragebogen (Qualtrics) auszufüllen und die als Video eingebetteten Übungen wie Kniebeugen, Wadenheben sowie Berührung der Zehen im Stand mit angewinkelten und mit gestreckten Beinen durchzuführen.

5- bzw. 7‑Punkt-Likert-Skalen sowie Intervall- und Nominalskalen wurden verwendet, um demografische Daten (Alter, Geschlecht, Beruf etc.), psychosoziale Faktoren (tägliche Aktivitätsmuster, Lebensstil, Schlafqualität, Ernährungsgewohnheiten etc.; [35]), medizinische Vorgeschichte (Operationen, Kniebeschwerden, Physiotherapieerfahrungen, allgemeiner Gesundheitszustand etc.; [36]), Persönlichkeitsmerkmale (Ten-Item-Personality-Measurement-Skala (TIPI); [37]) und das erwartete und wahrgenommene Anstrengungs‑/Schmerzniveau (Pain Numeric Rating Scale (NRS); [38]) zu bewerten. Die Teilnehmer*innen bewerteten zunächst jeweils die erwartete Anstrengung beim Betrachten der Videos der 4 Übungen, bevor sie aufgefordert wurden, die Übungen durchzuführen und danach die tatsächlich empfundene Anstrengung im Fragebogen einzutragen [39–41].

Insgesamt nahmen 116 Personen an der Studie teil, darunter 62 Frauen und 52 Männer. Der Datensatz enthielt vollständige Antworten aller Teilnehmer*innen, 60,3 % waren unter 25 Jahre alt (14,7 % 25–34 Jahre; 12,1 % 35–44 Jahre; 12,9 % über 45 Jahre). Alle trieben wöchentlich Sport, meist Spazierengehen, Wandern, Basketball oder Fußball. Sie gaben an, sich bei guter Stimmung mehr zu bewegen, berichteten am Studientag von positiver Stimmung und allgemein guter Gesundheit. 54 Teilnehmer*innen (46,6 %) hatten bereits mindestens einen chirurgischen Eingriff gehabt; davon hatten 26 Personen im Anschluss eine Physiotherapie erhalten. 26,9 % hatten sich vollständig an den Therapieplan nach der Operation gehalten. Die Auswertung zeigte, dass bei 40 % der Antworten Diskrepanzen zwischen der erwarteten und der tatsächlich empfundenen Anstrengung bei den Übungen bestanden, d. h., die Übungen wurden von Teilnehmer*innen als leichter oder schwerer empfunden als ursprünglich erwartet. Im Rahmen des digitalen Fragebogens hatten die Teilnehmer*innen die Möglichkeit, durch freiwillige Angabe ihrer E‑Mail-Adresse der Zusendung ihrer individuellen Studienergebnisse zuzustimmen. Im Anschluss an die Auswertung der Studie wurden die persönlichen Ergebnisse individuell per E‑Mail an die Teilnehmer*innen versendet.

### Training eines Basismodells für künstliche mentale Modelle

Für das Training des Basismodells mit den Daten der indirekten Beobachtung wurde ein mehrstufiger Prozess bestehend aus (1) Auswahl vortrainierter, maschineller Lernmodelle, (2) Evaluierung der Performanz der ausgewählten Modelle in Kombination mit den gegebenen Daten und (3) Feinjustierung (Fine-Tuning) der Modelle durchgeführt.

Für das Training wurden mit Llama‑2 (7B), Llama‑3 (8B), Llama‑3.1 (70B) und GPT‑4.o‑mini große, vortrainierte Large Language Models (LLMs) ausgewählt. Llama‑2 (7B), Llama‑3 (8B) und Llama‑3.1 (70B) sind LLMs von Meta, die Sprache verstehen und generieren können. Die angegebenen Ziffern stehen für die Größe des Modells – die kleineren Modelle (7B, 8B) sind schneller und benötigen weniger Rechenleistung, während das größere Modell (70B) tiefere und präzisere Antworten liefern kann. GPT‑4.o‑mini ist eine kompaktere Version von OpenAIs GPT‑4.o‑Modell, optimiert für Effizienz, aber immer noch in der Lage, komplexe Aufgaben wie Textgenerierung und Analyse zu bewältigen.

**Abb. 3:**
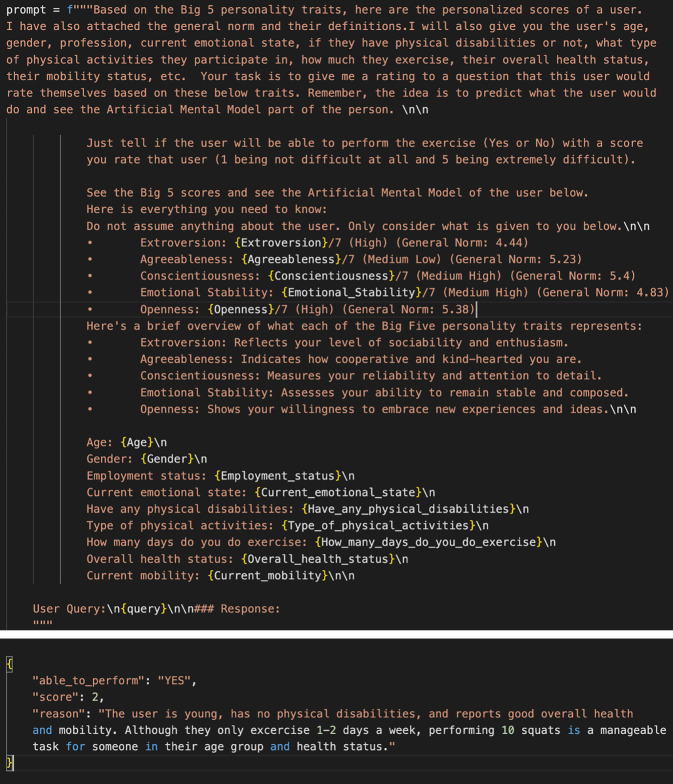
Beispiel für eine Anweisung (Prompt) an das Basismodell im Kontext der Feinjustierung (Fine-Tuning) mit Angabe von Daten der Person (z. B. Persönlichkeitsprofil, Alter, Geschlecht, Aktivität) sowie die Antwort des Modells (Response), d. h. dessen Vorhersage der voraussichtlichen Anstrengung, die Angabe des Pain-Scores für diese Person bei der Durchführung einer Übung (10 Kniebeugen) und die Angabe einer Begründung für die Vorhersage

Diese Modelle wurden in einem zweiten Schritt hinsichtlich ihres Vermögens evaluiert, für eine Person die erwartete Anstrengung einer körperlichen Übung (inkl. Pain-Score) vorherzusagen. Hierbei wurde Prompt Engineering genutzt. Ein Prompt ist die Eingabe oder Anweisung, die einem LLM gegeben wird, um eine bestimmte Antwort oder Aktion zu erhalten. Prompt Engineering ist die Kunst, diese Eingaben so zu gestalten, dass das Modell möglichst präzise und hilfreiche Antworten liefert. Die Prompts wurden unter Verwendung von Daten der Studie, wie z. B. demografischen Angaben und Persönlichkeitsmerkmalen der Teilnehmer*innen, ausgearbeitet (Abb. 3). Mithilfe der Prompts wurden die Modelle zur Vorhersage von voraussichtlicher Anstrengung, dem Pain-Score einer Person sowie der Angabe einer Begründung für die Vorhersage angeleitet. Dies erlaubte eine erste Bewertung der Leistung der Modelle.

Anschließend wurden die Modelle mit der besten Leistung, in diesem Fall Llama‑3 (70B) und Llama‑3 (8B), feinjustiert, d. h., ein Fine-Tuning wurde durchgeführt.^4^ Fine-Tuning ist ein Verfahren, bei dem ein bereits vortrainiertes maschinelles Lernmodell mit spezifischen neuen Daten weiter trainiert wird, um seine Leistung für eine bestimmte Aufgabe, wie in diesem Fall die Vorhersage von Annahmen einer konkreten Patient*in hinsichtlich zu erwartender Anstrengung und Schmerz in der Reha, zu verbessern. Dabei wird das vorhandene Wissen des Modells genutzt, sodass weniger Daten und Rechenleistung benötigt werden als beim Training von Grund auf. Basierend auf den Daten der Studie der indirekten Beobachtung wurde für das Fine-Tuning ein kuratierter, großer, synthetischer Datensatz erzeugt. Hierbei wurde darauf geachtet, jegliche Art von Datenverzerrung zu vermeiden, da dies zur Inzeption stereotyper, diskriminierender und irreführender Verhaltensweisen im Modell führen kann [42].

Um ein diskriminierungsfreies Basismodell zu trainieren, wurden Datenqualitätsprüfungen durchgeführt, indem z. B. die gleichmäßige Datenverteilung über demografische und verhaltensbezogene Faktoren analysiert wurde (u. a. Alter, Geschlecht, medizinische Vorgeschichte, Fitnesslevel). In einem weiteren Schritt wurde der synthetische Datensatz auf logische Inkonsistenzen überprüft, d. h., es wurde analysiert, ob der Gesundheitszustand logisch mit den Bewertungen der erwarteten bzw. tatsächlichen Schwierigkeit der Übungen korreliert. Identifizierte Unstimmigkeiten führten zu einer neuen Generierung der betroffenen synthetischen Stichproben. Das Vorgehen trug dazu bei, Verzerrungen bei der Generierung der synthetischen Daten zu verringern und sicherzustellen, dass der Datensatz die Vielfalt der Zielpopulation widerspiegelt.

Die synthetisch erzeugten Daten der Studie wurden anschließend als Prompts für das Fine-Tuning der Modelle genutzt. Nach Abschluss dieses Fine-Tunings wurden die Modelle schließlich auf Basis der realen Daten der Studie bewertet, um festzustellen, ob die Modelle in der Lage sind, für reale Personen die erwartete bzw. tatsächliche Schwierigkeit von Übungen vorherzusagen.

## Evaluierung des Basismodells

Ziel der Erhebungsphase war die Generierung eines diskriminierungs- und verzerrungsfreien, domänenspezifischen Basismodells eines KMM im Bereich der Knierehabilitation. Hierfür wurden unterschiedliche vortrainierte KI-Modelle, d. h. LLMs, trainiert und deren Leistung anhand von 2 Klassifikationsaufgaben bewertet: (1) Vorhersage, ob eine Person eine Übung durchführen kann oder nicht (binär, d. h. ja oder nein), und (2) Vorhersage des erwarteten Schmerzes (Pain-Score) bei einer Übung (NRS-Skala 1–5). Die Leistung wurde neben den Metriken Sensitivität und Spezifität u. a. durch die Accuracy (Genauigkeit) gemessen. Diese bezeichnet den Anteil der korrekt klassifizierten Beispiele an der Gesamtzahl der Beispiele und ist eine häufig verwendete Metrik zur Bewertung der Leistung eines maschinellen Lernmodells (Tab. 1).

**Tab. 1:** Evaluierung der vortrainierten Large Language Models (LLM) nach Feinjustierung und nach Entfernung von Verzerrungen und Halluzinationen auf Basis der Accuracy (Genauigkeit) bei der Vorhersage der Anstrengung bzw. des zu erwartenden Schmerzes einer körperlichen Übung, z. B. 10 Kniebeugen (Pain-Scores, NRS-Skala 1–5) für Personen mit einem bestimmten Profil (z. B. Persönlichkeit, Alter, Geschlecht, Gesundheitszustand; Kontext)

Vortrainiertes Large Language Model (KI-Modell)	Nach Feinjustierung (Fine-Tuning): Accuracy (%)	Nach Entfernung von Verzerrungen und Halluzinationen: Accuracy (%)
Llama‑3 (70B; Meta)	31,87	79,17
Llama‑3 (8B; Meta)	92,00	95,00

Das feinjustierte Llama-3-(8B-)Modell erreichte mit 92 % die höchste Accuracy bei der korrekten Vorhersage des zu erwartenden Pain-Scores, was seine Fähigkeit unter Beweis stellt, domänenspezifisches Wissen der Kniereha und den Kontext einer Person effektiv zu integrieren. Daraus lässt sich schließen, dass das Modell für eine spezifische Person mit einem bestimmten Profil in 92 von 100 Fällen vorhersagen kann, wie diese die Anstrengung bzw. den zu erwartenden Schmerz einer körperlichen Übung einschätzen wird. Im Gegensatz dazu zeigte das größere Modell Llama‑3 (70B) einen signifikanten Leistungsabfall bei der Feinabstimmung und erreichte nur 31,87 % Accuracy. Dies geht einher mit den Herausforderungen beim Fine-Tuning sehr großer Modelle, die insbesondere die Überanpassung der Modelle (Overfitting) bzw. verringertes Generalisierungsvermögen im Fall von begrenzten, domänenspezifischen Datensätzen betreffen.

Um sicherzustellen, dass die Modelle zu einem diskriminierungs- und verzerrungsfreien Basismodell eines KMM führen, wurden die Vorhersagen der Modelle eingehend überprüft. Bei Fällen falscher Vorhersagen wurde untersucht und evaluiert, ob diese Fehler überproportional Personen betrafen, die bestimmten demografischen Gruppen angehören, z. B. Geschlecht, Alter. Bei der Analyse wurden die Ergebnisse der Modelle nach demografischen Kategorien aufgeschlüsselt und Fehlerquoten für jede Gruppe berechnet. Die Fehlklassifizierungsparität, d. h. der Anteil falscher Vorhersagen für eine bestimmte Gruppe, wurde berechnet, um zu beurteilen, ob die Fehler zwischen den Gruppen variieren. Die Analyse des feinjustierten Llama-3-(8B-)Modells ergab, dass der Anteil der falschen Vorhersagen für männliche und weibliche Teilnehmer*innen 8,0 % bzw. 7,6 % betrug, was auf eine geringe geschlechtsspezifische Verzerrung hindeutet. Im Fall des Llama-3-(70B-)Modells war die Fehlerquote für Frauen (24,19 %) deutlich höher als für Männer (7,69 %), d. h., hier bestand eine demografische Ungleichheit bei den Vorhersagen des Modells. Um die Verzerrungen und Halluzinationen der feinjustierten Modelle zu reduzieren, wurde das Konzept der Konfidenzwahrscheinlichkeiten (*Confidence Probabilities*) genutzt. Diese beschreiben die Wahrscheinlichkeiten, mit denen ein Modell seine eigenen Vorhersagen trifft, und geben an, wie sicher es sich bei einer bestimmten Klassifikation oder Entscheidung ist. Hierfür wurde ein Hilfsmodell auf den Rohwerten (Logits) trainiert, die die Modelle als nicht normalisierte Wahrscheinlichkeiten jeder möglichen Ausgabe zuweisen, d. h. den möglichen Pain-Scores von 1 bis 5. Das Hilfsmodell nutzt die Vorhersagen der Modelle und analysiert darunterliegende Muster, um sie mit den realen Studienergebnissen abzugleichen.^5^ Die Auswertung verdeutlichte, dass die zusätzliche Anwendung eines Hilfsmodells, die Accuracy verbessert, indem Verzerrungen und Halluzinationen reduziert werden (Tab. 1). Die Accuracy des feinjustierten Llama-3-(8B-)Modells konnte von 92 % auf 95 % erhöht werden; im Fall des Llama-3-(70B-)Modells verbesserte sich die Leistung auf 79 %.

## Ausblick: Individualisierung des Basismodells

Das bereinigte und evaluierte Llama-3-(8B-)Modell entspricht der Zielstellung der Erhebungsphase im Projekt, ein diskriminierungs- und verzerrungsfreies, domänenspezifisches Basismodell eines KMM im Bereich der Kniereha zu generieren. Dieses Basismodell stellt die Ausgangsbasis für die nun folgende Individualisierungsphase dar (Abb. 2), in der das Modell für die Generierung von KMMs für individuelle Patient*innen in der Kniereha genutzt werden soll. Für das Training solch individueller KMMs sind kuratierte Daten wie Medikation, Rehatherapiepläne, Daten zu Operationsverfahren und -dauer, Komplikationen sowie nichtkuratierte Daten wie Interaktionen zwischen Patient*innen, Therapeut*innen und Ärzt*innen, Daten zu Bewegung, Schlaf, Fitnesszustand, Persönlichkeitsmerkmalen (Big Five), Schmerzkatastrophisierung, Selbstwirksamkeit etc. zu integrieren. Hierfür werden die Patient*innen 3 Wochen lang begleitet und in diesem Zeitraum vor und nach Physiotherapieeinheiten bezüglich ihrer Einschätzung zur erwarteten bzw. tatsächlichen Anstrengung bzw. Schmerz bei den körperlichen Übungen sowie dem empfundenen Fortschritt ihrer stationären Reha befragt (z. B. MPFL – „return to sport after injury“, Schmerz (VAS), Aktivität (Marx Activity Rating Scale, Tegener Aktivitäts-Score), Kinesiophobie (Tampa Scale of Kinesiophobia), Optimismus (Life Orientation Test-Revision)). Das resultierende, individuelle KMM wird in einem technischen Experiment sowie in einer empirischen Nutzer*innenstudie mit dem*der Patient*in und dem KMM evaluiert werden.

## Fazit

Die Rehabilitation (Reha) nach Knieverletzungen bzw. -operationen stellt für viele Patient*innen eine physische und psychologische Herausforderung dar. Trotz standardisierter Therapieansätze variieren die individuellen Erfolge erheblich. Kognitive Einschränkungen der Patient*innen und daraus resultierende unvollständige oder verzerrte Daten erschweren eine patient*innenzentrierte Versorgung. Hier stellt die Nutzung von künstlichen mentalen Modellen (KMM) in der Reha einen vielversprechenden Ansatz dar, um patient*innenzentrierte Therapien zu optimieren. Das Projekt FedWell zeigt, dass durch Modellierungen von KMMs mittels Künstlicher Intelligenz (KI) individuelle, physische, psychologische und umweltbedingte Einflüsse von Patient*innen besser erfasst werden können. Dies ermöglicht eine präzisere Vorhersage von Therapieerfolgen und eine personalisierte Anpassung der Rehamaßnahmen. Die Ergebnisse der in diesem Beitrag beschriebenen Erhebungsphase verdeutlichen das Potenzial von großen, vortrainierten KI-Sprachmodellen – *Large Language Models* – in ihrer Eignung als Basismodelle für KMM in der Rehabilitation.

Gleichzeitig zeigen die bisherigen Untersuchungen die Herausforderungen bei der Feinjustierung großer Sprachmodelle auf eine solch spezifische Domäne. Die Implementierung zusätzlicher Korrekturmechanismen erhöht die Vorhersagequalität und Diskriminierungsfreiheit der Modelle. Limitationen bestehen aufgrund der indirekt erhobenen und später synthetisch vergrößerten Daten, wodurch Abweichungen von realen Patient*innendaten möglich sind. Zudem ist die Validierung der Modelle bislang auf eine begrenzte Stichprobe beschränkt, was die Generalisierbarkeit einschränkt.

Zukünftig gilt es, das entwickelte Basismodell in klinischen Kontexten mit Patient*innen zu testen und zu validieren, um seine praktische Anwendbarkeit und Akzeptanz als KMM im medizinischen Alltag zu gewährleisten. Eine engere Verzahnung mit realen Patient*innendaten, insbesondere durch kontinuierliche Anpassung an individuelle Rehaverläufe, ist der nächste Schritt. Durch eine genauere Vorhersage von individuellen Herausforderungen können Behandlungspläne somit gezielt auf die Bedürfnisse einzelner Patient*innen zugeschnitten werden. Dies kann langfristig die Motivation der Patient*innen steigern und Therapieergebnisse verbessern.

## Footnotes

1 https://www.destatis.de/DE/Themen/Gesellschaft-Umwelt/Gesundheit/Vorsorgeeinrichtungen-Rehabilitationseinrichtungen/Tabellen/diagnosekapitel-geschlecht-vorsorge-reha.html (zugegriffen: 26.01.2025).

2 KMM reflektieren Annahmen, Erwartungen und Einstellungen des*der Patient*in über das Zielsystem, d. h. die Umgebung, mit der die Person interagiert. Dies unterscheidet sie von Digital Twins oder Nutzer*innen-Modellen, die ein digitales Abbild eines realen Objekts bzw. einer realen Person repräsentieren.

3 Halluzinationen bei LLMs treten auf, wenn das Modell überzeugend klingende, aber falsche oder erfundene Informationen generiert. Dies geschieht, weil das Modell nicht auf Faktenbasis denkt, sondern Muster in den Trainingsdaten extrapoliert, was zu Ungenauigkeiten oder falschen Schlussfolgerungen führen kann.

4 Die Vorhersagegenauigkeit des erwarteten Schmerzes (Pain-Score 1–5) betrug für GPT‑4.o‑mini 69,83 %, für Llama‑3 (8B) 83,26 % und für Llama‑3.1 (70B) 87,93 %. GPT‑4.o‑mini und Llama‑2 (7B; 12,07 %) überzeugten hier nicht, weshalb die beiden leistungsstärkeren Modelle für das Fine-Tuning ausgewählt wurden.

5 Das Hilfsmodell wurde als Regressionsmodell trainiert, um Logit-Werte auf die tatsächlichen Klassen des Pain-Scores 1–5 abzubilden. Dadurch erkennt es Muster von Über‑/Unterkonfidenz oder Unsicherheit und passt Vorhersagen entsprechend an. Eine stark konzentrierte Wahrscheinlichkeitsverteilung zeigt eine hohe Modellkonfidenz; eine flachere dagegen deutet auf Unsicherheit oder ein Verzerrungsrisiko hin. Durch die Anpassung liefert das Hilfsmodell kalibrierte, zuverlässigere Ergebnisse.
